# Nucleate boiling performance on nano/microstructures with different wetting surfaces

**DOI:** 10.1186/1556-276X-7-242

**Published:** 2012-05-06

**Authors:** HangJin Jo, SeolHa Kim, Hyungmo Kim, Joonwon Kim, Moo Hwan Kim

**Affiliations:** 1 Two-Phase Flow Lab, Department of Mechanical Engineering, Pohang University of Science and Technology (POSTECH), San 31, Hyoja-dong, Pohang, 790-784, Republic of Korea; 2 MEMS & Nanotechnology Lab, Department of Mechanical Engineering, Pohang University of Science and Technology (POSTECH), San 31, Hyoja-dong, Pohang, 790-784, Republic of Korea; 3 Two-Phase Flow Lab, Division of Advanced Nuclear Engineering, Pohang University of Science and Technology (POSTECH), San 31, Hyoja-dong, Pohang, 790-784, Republic of Korea

**Keywords:** nano/microstructure, nucleate boiling heat transfer, critical heat flux, surface wettability, capillary effect

## Abstract

A study of nucleate boiling phenomena on nano/microstructures is a very basic and useful study with a view to the potential application of modified surfaces as heating surfaces in a number of fields. We present a detailed study of boiling experiments on fabricated nano/microstructured surfaces used as heating surfaces under atmospheric conditions, employing identical nanostructures with two different wettabilities (silicon-oxidized and Teflon-coated). Consequently, enhancements of both boiling heat transfer (BHT) and critical heat flux (CHF) are demonstrated in the nano/microstructures, independent of their wettability. However, the increment of BHT and CHF on each of the different wetting surfaces depended on the wetting characteristics of heating surfaces. The effect of water penetration in the surface structures by capillary phenomena is suggested as a plausible mechanism for the enhanced CHF on the nano/microstructures regardless of the wettability of the surfaces in atmospheric condition. This is supported by comparing bubble shapes generated in actual boiling experiments and dynamic contact angles under atmospheric conditions on Teflon-coated nano/microstructured surfaces.

## Background

Boiling is a general mechanism in heat transfer systems, such as those used to cool electronic devices and power plant systems. In boiling, the two most important parameters are (1) the boiling heat transfer (BHT), which is directly related to the efficiency of a thermal device, and (2) the critical heat flux (CHF), which requires a safety limitation for the system. Therefore, to transfer or dissipate high heat flux from heat sources in real-world applications, thermal devices and systems should have high BHT and CHF. Over the past century, many techniques for enhancing BHT and CHF have been developed. Most recently, boiling experiments with treated surfaces have been used extensively to study the effect of heating surface characteristics on BHT and CHF.

Of the many surface characteristics, wettability and surface geometry are the key parameters for determining boiling performance. By affecting the dynamics of the phase interface adjacent to the heating surface, wettability and surface geometry influence overall nucleate boiling phenomena, from activated nucleation sites to CHF. In particular, a number of researchers have reported CHF enhancement on well-wetted surfaces [1-3]. The effect of wettability on CHF has also been confirmed by pool boiling experiments with nanofluids, using surfaces modified by nanoparticle deposition, which have recently drawn considerable attention due to the striking CHF enhancement obtained even with very low nanofluid concentrations [4,5]. Finally, the effect of wettability on CHF is reflected in Kandlikar’s theoretical CHF model, which includes a dynamic contact angle term [6]. Cavity geometry is also significantly related to boiling performance, especially in terms of activated nucleate site density, which is directly related to BHT enhancement. The shape or roughness of the microstructures determines the activated nucleate site density [7-9]. Moreover, microstructures also influence the CHF [10,11]. However, there are still obstacles to the realization of very high heat generation in thermal devices and systems.

The recent introduction of nanostructured surface modification techniques has opened a new chapter in the study of boiling phenomena and the development of very high heat transfer systems. The fabricated nanostructures provide greatly enhanced boiling performance [12-16]. In particular, reinforced capillary pumping of the working fluid onto the heated surfaces of nanostructures is the main contributor to an abnormally high CHF [13,17]. Kim et al. [12] used a ZnO surface to fabricate micro-, nano-, and micro/nanostructured surfaces and reported CHF improvement on these surfaces. Chen et al. [13] conducted pool boiling experiments on Si and Cu nanowires and reported dramatically enhanced CHF and BHT on the nanostructures. Ahn et al. [17] used liquid spreading phenomena to analyze the high CHF on zircaloy-4 nanostructured surfaces. However, even with these innovative studies, it has not been possible to draw any general conclusions on the performance of nanostructures in boiling systems, due to the lack of experimental boiling data on nanostructures with various surface characteristics. In particular, previous studies of boiling phenomena on nanostructures have focused exclusively on hydrophilic surfaces. Such biased reports in wettability could cause misunderstanding to the analysis of boiling phenomena because of complicatedly coupled surface factors in one boiling phenomenon: the effect of surface structure and wettability [18,19].

In this research, we conducted pool boiling experiments on hydrophilic and hydrophobic nanostructured surfaces with identical nanostructures to classify the wetting and the surface structural effect on nanostructures. The nanostructures (black silicon) were fabricated via a surface treatment procedure to create different wetting surfaces (super-hydrophilic and super-hydrophobic) with exactly the same surface structure and were used as heating surfaces to study boiling phenomena and performance. The results are expected to provide an important contribution in distinguishing the effects of surface structure and wetting on the basic mechanisms of BHT and CHF enhancement due to nano/microscale structures with totally different wetting characteristics.

## Methods

### Surface fabrication methods (black silicon)

The specimens were designed for both micro- and nanoscale structures, with a heater on the backside. To form the microstructures, an anisotropic wet-etching technique was used. The nanostructures were then fabricated on the microstructures by deep reactive-ion etching (DRIE). The presence of microstructures with sloped rather than vertical sidewalls is an important condition to ensure conformal formation of nanostructures. In order to fabricate microstructures with sloped sidewalls, a (100) silicon (Si) wafer was selected, owing to its unique sloped sidewall profile during the wet-etching process. A layer of thermally grown SiO_2_ was then formed on the wafer, and the top layer was patterned, using a photolithographic technique, as an etching mask for microstructure formation. Tetramethylammonium hydroxide was used to etch the exposed area of the (100) Si wafer for 12 min at 90 °C. After the microstructures were formed, the Si etching mask was removed. A 20-nm Ti layer and a 150-nm Pt layer were then deposited and patterned on the backside of the wafer for Joule heating. Nanograss structures were then fabricated by DRIE (specifically, the black silicon method). Conformal formation of silicon nanograss structures was made possible by the sloped sidewalls.

Additional surface treatments were used to realize different wettabilities on the same surface structure. Two surface treatment techniques were used in this research. The first of these was the O_2_ plasma technique, which was used to clean up some of the residue from the DRIE procedure and accelerate the growth of the native oxidation layer on the silicon surface. Due to the effect of the plasma, the exposed surfaces became super-hydrophilic for a few days. However, even though the effect of the plasma exposure was eliminated after a few days, the treated surfaces retained super-hydrophilic characteristics because of the mixed effect of natively hydrophilic silicon on surface smoothness and roughness [18,19]. In this study, we will refer to this type of treated surface as an ‘oxidized silicon nano/microstructured surface’ to indicate the presence of nano/microstructures with a native oxide layer on the silicon surface. The second surface treatment technique was used to create a hydrophobic surface on the nano/microstructures, by applying a Teflon coating to the plasma-exposed nano/microsurfaces. Robust Teflon-coated surfaces were fabricated via a spin coating procedure and a soft baking procedure at 90 °C for 10 min. This type of treated surface is called a ‘Teflon-coated nano/microstructured surface’ in this paper.

**Figure 1:**
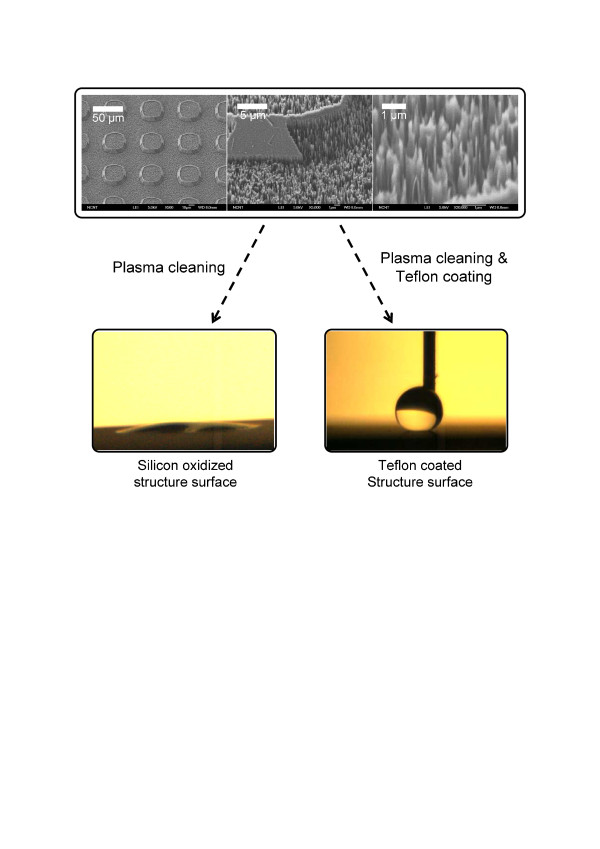
**SEM image and apparent static contact angle. **SEM image of the fabricated structured surface and apparent static contact angle on oxidized silicon and Teflon-coated nano/microstructures.

Detailed scanning electron microscopy (SEM) images and measurement results for the contact angles are shown in Figure 1. The sloped sidewalls of the microstructures promoted the conformal formation of nanograss structures via the black silicon method. The microstructures had a 30-μm gap and a 7-μm height. The nanograss diameter was on the scale of tens of nanometers. As Figure 1 shows, the oxidized silicon nano/microstructured surface had a 0° static contact angle. The static contact angle of the bare oxidized silicon surface was 54°. On the other hand, for the Teflon-coated nano/microstructured surface, the static contact angle was 162°, and for the bare Teflon-coated surface, the static contact angle was 123°. All contact angles were measured at room temperature under atmospheric conditions.

## Results and discussion

The test apparatus was designed to carry out pool boiling experiments under atmospheric pressure via the electrical Joule heating method, using an HP Agilent 6575A DC power supply (Santa Clara, CA, USA). The main test pool was an octagonal aluminum bath (with a capacity of 3 L) and was maintained in a saturated condition by a proportional-integral-derivative temperature controller. A high-speed camera (Redlake MotionXtra HG–100 K, San Diego, CA, USA) was installed on the visualization glass to capture images of the bubble dynamics in the nucleate boiling regime. To facilitate both heating and surface modification, a thin-film heater was embedded on one side of the silicon wafer, and artificial surfaces were created on the other side of the wafer via microelectromechanical system techniques. Taking all instrument errors into account, the maximum uncertainties of the heat flux, wall superheat, and heat transfer coefficient were estimated to be less than 15 kW/m^2^, 1.5 °C, and 0.56 kW/m^2^ °C, respectively, over the expected CHF range [20]. A numerical simulation was conducted to incorporate the effects of heat dispersal and heat loss. Based on the measured bottom heat flux and temperature information, the reduction procedure was repeated with several heat transfer coefficients until the calculated values matched the experimental magnitudes. The heat transfer coefficient was determined via numerical simulation, by finding a value that satisfied the experimentally obtained target condition.

Before experimenting with fabricated nano/microstructured surfaces, experiments were conducted on bare oxidized silicon and Teflon-coated surfaces (i.e., without any structures), and the results were analyzed to re-establish baselines. The wetting characteristics of a heating surface affect the overall nucleate boiling mechanism, including CHF and BHT, by influencing generated bubble dynamics on the surface. Good wettability leads to a higher CHF than poor wettability [1,6,20]. Based on this tendency, Kandlikar postulated a mechanism for CHF that includes the wettability effect [6]. In this study, the CHF values for bare oxidized silicon and Teflon-coated surfaces were found to be 786.23 and 178.98 kW/m^2^, respectively. A difference of this magnitude between hydrophilic and hydrophobic surfaces is consistent with the experimental tendencies found in the literature for various wetting surfaces.

**Figure 2:**
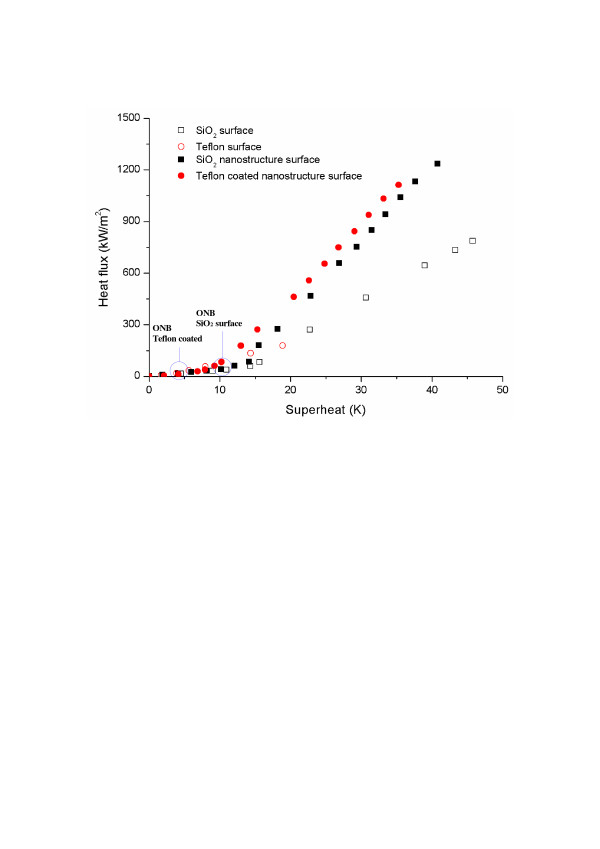
Comparison of boiling curve on each of the different heating surface conditions.

Wettability also significantly influenced BHT, by altering a number of bubble dynamics phenomena. First, each of the different wetting characteristics yielded a different starting point for nucleate boiling, which is known as the onset of nucleate boiling (ONB). In accordance with previous reports, the hydrophobic surface (contact angle greater than 90°) exhibited an earlier ONB than the hydrophilic surface since the free energy required to nucleate a bubble is less for a hydrophobic surface than for a hydrophilic surface [20-23]. This early ONB is one of the important features that explain BHT enhancement on a hydrophobic surface. Furthermore, wettability can also affect bubble growth and the departure mechanism. In the hydrophilic case, the direction of the surface tension at the triple point (three-phase intersection) is toward the generated bubble side. In the hydrophobic case, however, the direction of the surface tension is toward the outside. Therefore, for any given amount of generated vapor, the hydrophobic heating surface will have greater surface tension along the triple line (slowing the departure of bubbles from the surface) since it has a larger contact area with the bubble base than the hydrophilic surface. This is the origin of the differing bubble dynamics of hydrophobic and hydrophilic surfaces. Here, the hydrophobic surface exhibited larger and slower bubble generation than the hydrophilic surface, as well as chain bubble generation without a waiting period [20,23]. In view of these mechanisms, if no surface structures are present, the hydrophobic surface is expected to realize better BHT, with early nucleation and continuous generation of large bubbles in the low heat flux regime. However, in the high heat flux regime, the hydrophobic surface was unable to sustain a good boiling performance because too many large bubbles were generated, resulting in low CHF, as shown in Figure 2 and in accordance with previous reports [20,22].

The boiling characteristics of the oxidized silicon and Teflon-coated nano/microstructured surfaces were investigated and then compared with those of the bare oxidized silicon and Teflon-coated surfaces, as shown in Figure 2. According to previous research, simultaneous realization of high CHF and improved BHT is difficult using a normal homogeneous wettability surface (a hydrophobic surface is advantageous for BHT, but not for CHF, whereas the reverse is true for a hydrophilic surface). However, in the present study, the fabricated nano/microstructures exhibited enhancement of both CHF and BHT, independent of their wetting characteristics. This was consistent with the requirement for optimized heating surfaces in real-world applications. The increments of CHF and BHT on the different wetting surfaces were dependent on the surface wettability. In the literature, CHF shows a tendency to increase as the wetting characteristics of the heating surface improve. However, the CHF values for the fabricated oxidized silicon and Teflon-coated nano/microstructured surfaces were 1,236.09 and 1,112.33 kW/m^2^, respectively, constituting respective improvements of 57.2 % and 41.5 % over the bare SiO_2_ surfaces, even though the measured static contact angle of the Teflon-coated nano/microstructured surface was larger than that of the bare oxidized silicon surface. To demonstrate the effect of wettability, the CHF values obtained in this research were compared with Kandlikar’s CHF predictions for an infinite plate, which include the effect of wettability in the CHF mechanism. Since the predicted CHF values did not match the experimental values (due to the heater size effect), Kandlikar’s CHF predictions were modified by multiplying by a constant factor to fit the experimental data for the bare oxidized silicon and bare Teflon-coated surfaces, as follows:

(1) q″CHF=F×ifvρv0.51+cosθR162π+π41+cosθRcosφ0.5σgρf−ρv0.25

**Figure 3:**
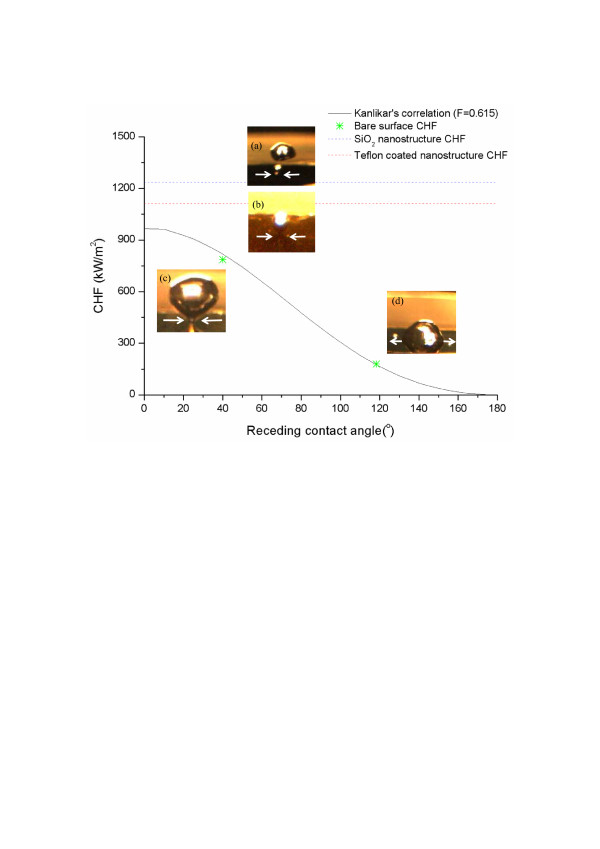
**Comparison between Kandlikar’s prediction [6] and experimental CHF and bubble dynamics in real boiling condition. **(**a**) SiO_2_ nanostructured surface, (**b**) Teflon-coated nanostructured surface, (**c**) SiO_2_ bare surface, and (**d**) Teflon bare surface.

Here, *F* is a constant factor (0.615 in this paper), *i*_fv_ is the latent heat, *ρ*_v_ and *ρ*_f_ are the vapor and fluid densities, respectively, *θ*_R_ is the dynamic receding contact angle, *φ* is the oriented angle of the heating surface, *σ* is the surface tension, and *g* is the gravitational acceleration. As Figure 3 shows, Kandlikar’s CHF predictions fit the bare SiO_2_ and Teflon surfaces well, but underestimated the CHF for the fabricated nano/microstructured surfaces, irrespective of the heating contact angle.

Such highly enhanced CHF values on nanostructures have been reported in a number of recent studies. Chen et al. [13] carried out pool boiling experiments on Si and Cu nanowires and observed respective CHF increases of 132.88 % and 139.04 % over a plain Si surface. In the work of Kim et al. [12], the reported CHF improvement ratios on microstructures, nanostructures, and nano/microstructures ranged from 47.37 % to 107.49 %. The significant CHF improvement on fabricated nanostructured surfaces can be elucidated by considering the effect of capillary phenomena, which carry cooler working fluid into the hot spots to rewet them and delay CHF occurrence [24,25]. Ahn et al. [17] observed CHF improvements ranging from 17.63 % to 55.78 % on nanostructures and nano/microstructures and analyzed the different CHF enhancement ratios on surfaces where capillary dispersal occurred, by combining Kandlikar’s wetting consideration and the capillary spreading rate effect. Such variance of CHF increment on nanostructured surfaces is caused by the topological effect of the nanostructures on capillary phenomena [17]. Consequently, it is certain that the liquid dispersal and hot-spot cooling mechanism provided by capillary phenomena is a dominant factor in the higher CHF values observed on nanostructured surfaces (compared to plain surfaces). Here, the dispersal phenomena were confirmed on the oxidized silicon nano/microstructured surface via a surface characterization procedure (the measured contact angle being 0°), together with their beneficial effect on the CHF in this case. However, CHF enhancement on a Teflon-coated nano/microstructured surface cannot be directly explained in terms of capillary wicking phenomena since the dispersal characteristics are absent with a very high contact angle.

We now propose a plausible mechanism to explain the significantly enhanced CHF on the Teflon-coated nano/microstructures as well as the CHF difference between the oxidized silicon and Teflon-coated nano/microstructured surfaces. According to a previous X-ray scattering study, water is able to penetrate 5 to 10 nm into nanoscale hydrophobic cavities, independent of cavity depth, when a hydrophobic nanostructure is immersed in water [26]. This means that the contact angle on a hydrophobic nanostructure under actual boiling conditions can be dramatically reduced from the dynamic contact angle measured under dry conditions since it will be affected by water seeping into the hydrophobic nanostructures. We can confirm this phenomenon by comparing our visualization results, which include the difference between the dynamic contact angle of a water droplet on a dry surface in air (Figure 1) and that of a surface wetted by surrounding water under actual boiling conditions (Figure 3). According to Kandlikar’s prediction, the main mechanism of enhanced CHF with increasing wettability is related to the generated bubble shape under actual boiling conditions. Hence, enhancement of CHF with reduction of the contact angle of generated bubbles on a heating surface is consistent with Kandlikar’s prediction. Nevertheless, even when we include the effect of the generated bubble shape, the dramatically enhanced magnitude on the Teflon-coated nano/microstructured surface cannot be described solely in terms of the wettability effect, as Figure 3 indicates.

As was previously mentioned, capillary phenomena have already been acknowledged as the main reason for CHF enhancement on nano/microstructured surfaces. Thus, the additional CHF increment on the Teflon-coated nano/microstructures might have been caused by capillary phenomena fueled by penetrating water. Based on the earlier report on hydrophobic nanostructures cited above, the water penetration depth should have been 5 to 10 nm, even though the depth of the nanocavities was over 100 nm. However, 5 to 10 nm of penetration seems inadequate to produce such a high CHF on a Teflon-coated nano/microstructured surface since such a value is not much different from the roughness of a cleaned surface. The degassing procedure (for removing dissolved gas from water), which was conducted before the main experiment, appears to play an important role in resolving this issue. This procedure induces some of the nanobubbles to coalesce and be detached from the surface by buoyant forces [27]. Therefore, after the degassing procedure, water will have a better chance of penetrating deeper than 5 to 10 nm into the vacancy left by the detached nanobubbles. (Of course, it cannot completely fill the nanocavities since degassing cannot remove all nanobubbles from the nanostructure.) Accordingly, it is our hypothesis that the combined effects of degassing and water penetration into the nano/microstructures via capillarity contributed to the enhanced CHF on the Teflon-coated nano/microstructures.

Furthermore, it can be hypothesized that differing capillary phenomena on structured surfaces with different wettabilities caused the CHF difference between the oxidized silicon nano/microsurfaces and the Teflon-coated nano/microsurfaces. According to the bubble dynamics visualization results shown in Figure 3, the apparent contact angles of oxidized silicon and Teflon-coated nano/microstructures were almost the same under actual boiling conditions. Hence, capillary phenomena are the only remaining factor. Decisively, the total amount of water absorbed into the nanostructures was clearly different for the two cases. The water absorbed by the hydrophilic nanostructures was able to penetrate into the bottom of the nanocavities, which was not possible in the hydrophobic nanostructures because of the existence of nanobubbles [26,28]. In conclusion, the CHF difference between the oxidized silicon and Teflon-coated nano/microstructures was apparently caused by the difference in capillary pumping capacity, which in turn is related to the presence of nanobubbles on the hydrophobic nanostructured surfaces.

**Figure 4:**
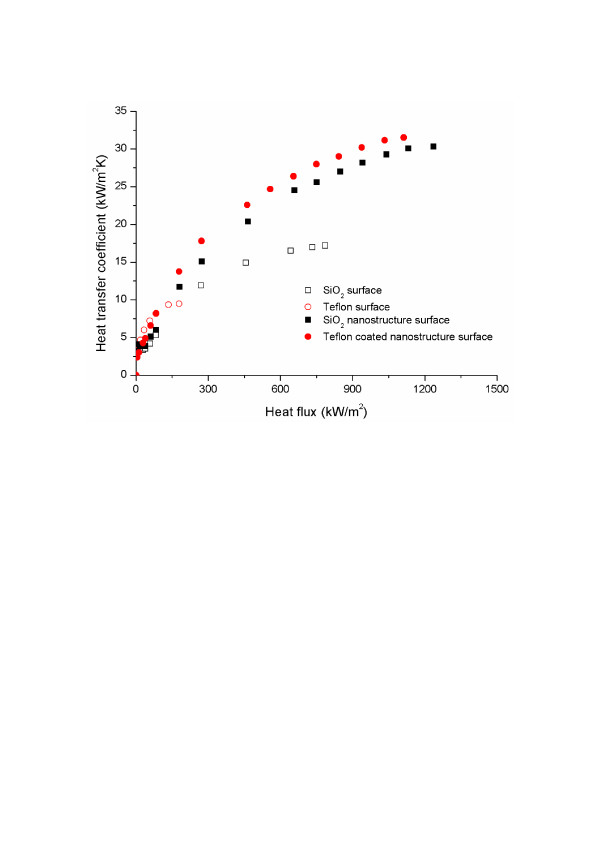
Comparison of BHT coefficient on each of the different heating surface conditions.

**Figure 5:**
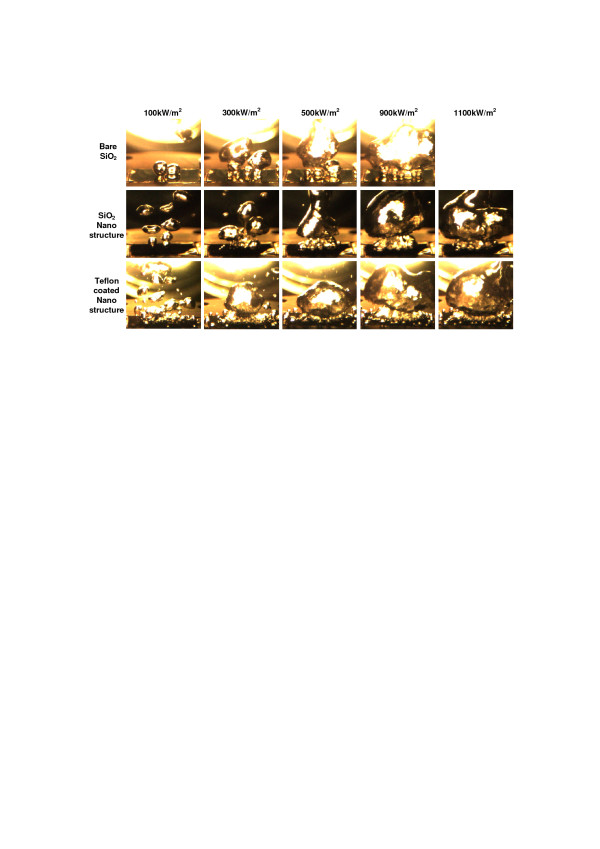
Bubble dynamics on each of the different heating surface conditions at different heat flux conditions.

The fabricated nano/microstructured surfaces also showed improved BHT. Near the CHF, the BHT values for the fabricated oxidized silicon and Teflon-coated nano/microstructures were 30.31 and 31.52 kW/m^2^·K, respectively, yielding 76.4 % and 83.5 % enhancements of the BHT result for bare oxidized silicon (17.18 kW/m^2^·K). To investigate BHT augmentation on fabricated surfaces at a given heat flux, a BHT comparison was conducted as shown in Figure 4, and enhanced BHT was observed on the fabricated surfaces. This enhanced BHT is provided by increasing the activated nucleate site density; as Figure 5 shows, there are numerous activated nucleate sites on the fabricated nanostructured surfaces. This phenomenon is consistent with previous experimental boiling results on nanostructured surfaces [13-15]. In particular, Chen et al. [13] also obtained higher BHT on nanostructured surfaces than on a plain surface (by 127.27 % to 132.95 %). However, the reason behind it is still being debated since the nanostructures or cavities are not expected to be activated at low superheat. The specimens used in this research were designed for both micro- and nanoscale structures, rather than just nanostructures. However, since there were no micron-sized vacancies or cavities on the fabricated nano/microstructured surfaces (as Figure 1 shows), we are faced with the same problem as that of the previous studies. The debate is focused on several factors.

**Figure 6:**
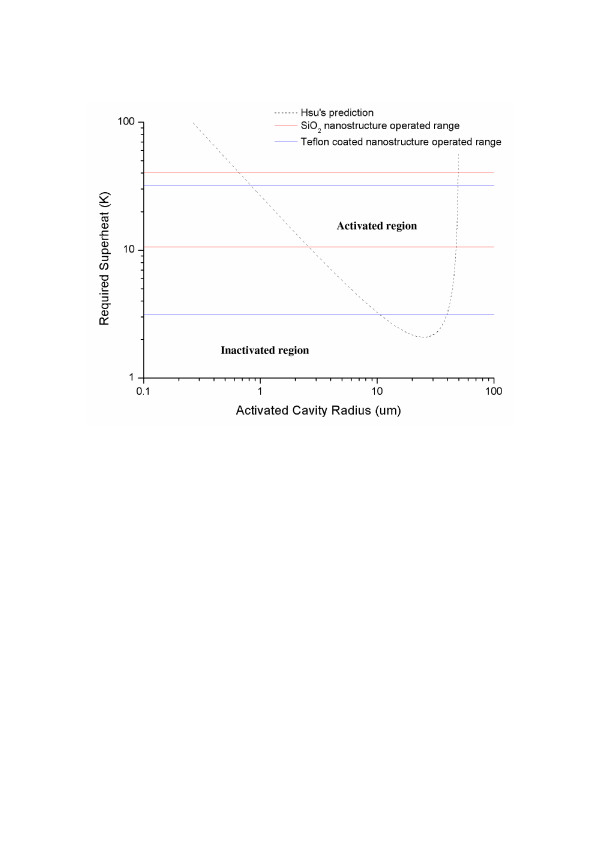
**Comparison between Hsu’s prediction [29]and actual activated range in real boiling condition.**

One of these is nanobubbles. Nanobubbles are conjectured to provide a seed vapor for creating numerous activated bubbles. However, a nanobubbles hypothesis is only supported on hydrophobic surfaces, and not on hydrophilic surfaces, since only hydrophobic surfaces can have nanobubbles. The other candidate is micrometer defects. Even if a nanostructured surface is evenly fabricated, it can still have micron-sized defects, owing to the limitations of the manufacturing technique. To reliably determine the size of these microscale defects, the possible activated cavity size was calculated on the basis of Hsu’s prediction, which was developed for that purpose [29]:

(2) $$r_{\max,\min}=\frac{\delta_{t}}{4}\left[1\pm \sqrt{1-\frac{12.8\sigma T_{\mathrm{sat}}}{\rho_{v}i_{\mathrm{fv}}\delta_{t}T_{\text{super}}}}\right]$$

where *r*_max,min_ denotes the activated maximum and minimum cavity radii, *δ*_t_ is the thermal layer thickness (which was assumed to be 0.1 mm in this paper), *T*_sat_ denotes the saturated temperature, and _super_ indicates a superheated condition. According to Hsu’s analysis, the activated cavity size ranges for the oxidized silicon and Teflon-coated nano/microstructured surfaces near the ONB were 2.6 to 47.4 and 10.6 to 39.4 μm, respectively, and their ranges were extended to 0.7 to 49.4 and 0.8 to 49.2 μm, respectively, as the superheat of the heating surface increased to the CHF (Figure 6). This means that if the size of the micron defects of the fabricated nanostructures was approximately 1 to 40 μm, they could have been the cause of the extensive bubble nucleation. Nevertheless, some doubts remain. To create a large number of bubbles in this way, a fabricated surface should have as many micron defects as activated sites. However, the presence of so many micron defects was not intended or ensured by the nanostructure fabrication procedure. Thus, there is a continuing debate on the mechanism of high BHT on nanostructures. Now, to improve the discussion of the reason why there are numerous activated nucleate sites on nanostructured surfaces, we have conjectured that the existence of numerous bubbles on nanostructures is caused by the morphological characteristics of the nanostructures. If liquid molecules are present on a concave portion of the nanostructures, the liquid will be more easily heated by the surrounding solid rod of nanostructures. The point is that the amount of the liquid on a concave portion of the nanostructures is not much as on a plain surface, but the exposed surface area of the nanostructured surface to liquid is much wider than that of the plain surface because of the small vacancy between many nanostructures. In other words, owing to the heat-focusing capability of water in a nanoconcave portion, the heat distribution adjacent to the heated surface on a nanostructured surface might be changed compared to that on a plain surface, and it could be related with greater bubble nucleation on a nanostructured surface than on a plain surface under a given heat flux condition. Therefore, we hypothesize that the change of heat distribution adjacent to nanostructures, induced by morphological characteristics, contributes to more extensive bubble generation and enhances the BHT on nanostructured surfaces. Even though the mechanism still has many calculations and experimental proofs which are required to be specified, such inspiration will contribute to improve the discussion of the mechanism of BHT enhancement on nanostructured surfaces. This hypothesis will be the subject of future research, based on a better fabrication technique and a fresh inspiration.

The important point taken from the present experimental study is that BHT is dramatically enhanced on a nanostructured surface (compared to a plain surface), regardless of wettability, and more enhanced on a Teflon-coated nano/microstructured surface than on an oxidized silicon nano/microstructured surface. The superior enhancement of BHT on Teflon-coated nano/microstructures is related to the occurrence of the ONB. As was previously mentioned, the ONB occurs earlier on a hydrophobic surface than on a hydrophilic surface. In this study, the ONB for the bare Teflon-coated surface was 4.23 K, earlier than the ONB for the bare oxidized silicon surface (10.16 K). Interestingly enough, this tendency also appeared on the fabricated nano/microstructured surfaces. The ONB values of the Teflon-coated and oxidized nano/microstructures were 3.16 and 10.05 K, respectively, which are virtually the same as the values for the corresponding bare surfaces. This means that the wettability effect, and not the surface structure effect, mainly determined the ONB of each heating surface. As Figure 2 indicates, this ONB variation on surfaces with different wettabilities caused the overall difference in BHT between the Teflon-coated and oxidized nano/microstructured surfaces. Consequently, it can be inferred that nanostructures induce a high BHT, due to their numerous activated nucleate sites, while wettability has a small effect on BHT when the structures are unchanged, owing to the different starting points of the nucleate boiling regime (ONB).

## Conclusions

We conducted a pool boiling experimental study on identical nano/microstructures with different wettabilities to examine the effect of wettability and surface structure on BHT and CHF. The results indicated dramatically enhanced BHT and CHF on nano/microstructures, independent of their wettability. However, the BHT and CHF increments were affected by the surface wettability. The Teflon-coated nanostructured surfaces had lower CHF and better BHT than the oxidized silicon nanostructured surfaces. We have conjectured that the combined effect of surface wettability and capillary wicking contributes to CHF improvement, even though the measured contact angle under dry conditions was very high, since the dynamic contact angle of the Teflon-coated surface in an actual boiling situation could be sharply reduced by water penetrating into the nanoscale vacancies left by detached nanobubbles after the degassing procedure. As a result of this study, the effect of surface structure and wettability on high BHT is also confirmed. By analyzing the visualization results for boiling phenomena on the fabricated structures, we identified numerous activated nucleation sites, which were responsible for the BHT improvement. The difference in BHT on the same nanostructures with different wettabilities was caused by the difference in the ONB. Therefore, the activated nucleate site density, resulting from the surface structure, and the variation of the ONB with wettability together determined the point of initiation of overall BHT enhancement on the nanostructures.

## Competing interests

The authors declare that they have no competing interests.

## Authors’ contributions

The work conducted here was collaborated with all authors. HJJ designed and analyzed the boiling experiments and drafted the manuscript. SHK carried out and analyzed the pool boiling experiments. HK and JK fabricated the surface used in this study via MEMs technique. MHK governed all procedures and conceived the research theme as the corresponding author. All authors read and approved the final manuscript.
